# Accident from Ignition of Ether

**Published:** 1917-08

**Authors:** 


**Accident From Ignition of Ether.** The Pacific Med. Jour., June, reports an explosion from a spark in a parabolic electric head-light. The name of the surgeon is withheld but it is said that he can readily be identified if seen and, from the lack of a note of mourning in the account, we infer that the results were not serious.

